# Towards Carbon Neutrality: Carbon Emission Performance of Science and Technology Finance Policy

**DOI:** 10.3390/ijerph192416811

**Published:** 2022-12-14

**Authors:** Hong Xu, Baozhen Liu, Kai Lin, Yunyun Zhang, Bei Liu, Mingjie Xie

**Affiliations:** 1 Business School, Shandong Normal University, Jinan 250358, China; 2 School of Management, Nanjing University of Posts and Telecommunications, Nanjing 210003, China

**Keywords:** science and technology finance policy, carbon emission, difference-in-differences, interactive effect, radiation effect

## Abstract

Combining technology with finance is the focus of supporting economic structure adjustment, and environmental benefits are also the proper meaning of the policy. Using the panel data of 274 cities in China from 2006 to 2017, this paper examines the impact of the Science and Technology Finance Policy (STFP) on carbon emission intensity in pilot cities and the transmission mechanisms through the difference-in-differences method and further explores the impact of STFP on the carbon emission intensity in neighboring cities. The results show that (1) STFP has significantly reduced carbon emission intensity in pilot cities and has dynamic effects, which gradually increase over time. There is significant heterogeneity in the carbon emission reduction effect of STFP, which produces stronger policy effects in first and second-tier cities and cities with higher information levels. (2) STFP achieves carbon emission reduction effects through three main pathways: the total factor productivity improvement effect, innovative elements agglomeration effect, and industrial structure optimization effect. (3) The STFP and national e-commerce demonstration policy have an interactive effect, and the two jointly contribute to the reduction in carbon emission intensity. From the perspective of a spatial effect, STFP has a radiation effect; that is, STFP not only reduces local carbon emission intensity but also curbs the carbon emission intensity in neighboring areas.

## 1. Introduction

**Figure 1:**
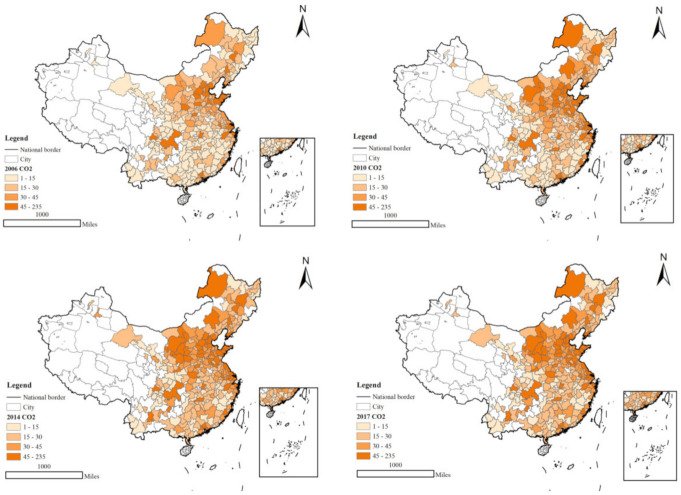
China’s carbon emission trends in 2006, 2010, 2014, and 2017.

At the Paris climate change conference, China promised to achieve a 40%-45% reduction in carbon intensity by 2020, while the *2020 China Ecological and Environmental Status Bulletin* states that the CO_2_ emission intensity per unit of GNP in 2020 decreased by 18.8% compared with 2015. In the same year, China’s total coal consumption reached 56.8%, still occupying a considerable proportion, and this high energy dependence is the main reason for the sharp CO_2_ emissions [1,2,3]. As shown in Figure 1, CO_2_ emissions showed a rapid upward trend during 2006–2017, and the trend of CO_2_ emissions in the north was more obvious due to problems such as coal burning in the winter. It can be seen that China needs to explore more ways to reduce carbon emissions and actively respond to climate change in order to achieve carbon neutrality by 2060. Today the world is experiencing a century of great and unprecedented change, and COVID-19 has seriously damaged the world’s economic development [4,5], so China faces huge challenges in the new development stage. At present, regions have insufficient innovation power, have arduous tasks of ecological and environmental protection, and contradictions of unbalanced and insufficient development are increasingly emerging, which do not meet the requirements of high-quality development [6,7]. A rational and complete financial system helps promote physical capital accumulation and economic growth [8], and public R&D funds that support high-tech industries will also guide private R&D investment in such industries [9]. The government should encourage financial institutions to provide loan concessions for high-tech supply chains, such as carbon-emitting technologies, and promote clean energy technologies, which can have a positive impact on curbing carbon emissions [10]. In the era of rapid information development, technology and finance have become the main focus of economic development, and solving financing problems for the development of high technology should be given high priority.

**Figure 2:**
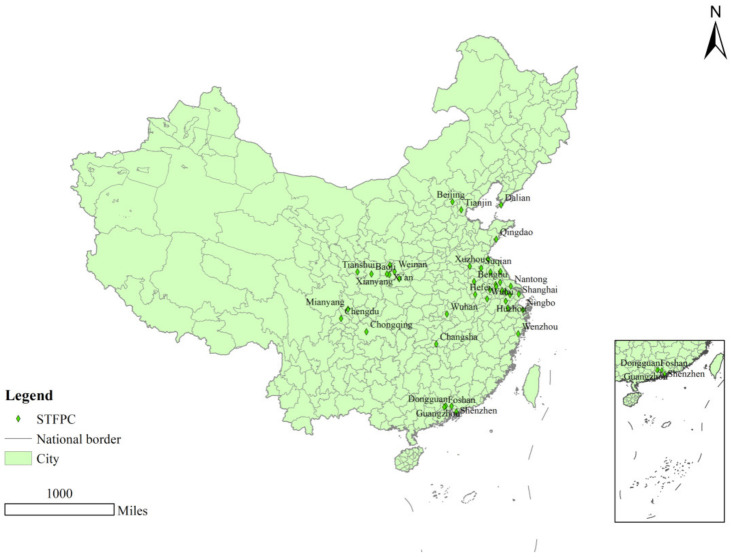
The distribution of science and technology finance pilot cities in China.

The financial innovation system, as the foundation of technological innovation systems, is an important driver of innovation activities [11], helping to alleviate financial pressure and reduce energy intensity, and the coordinated promotion of financial and environmental policies changes the direction of technological progress [12]. Both financial development and technology innovation contribute to economic growth [13,14]. While promoting rapid economic growth, financial development not only drives innovation in energy technology but eases the contradiction between economic development and energy consumption [15] and also helps improve environmental quality [16]. The *Several Opinions on Promoting the Integration of Science, Technology, and Finance to Accelerate the Implementation of Independent Innovation Strategy* released in 2011 helps to guide the allocation of financial resources to the field of technology, which is a solid foundation and guarantees the cultivation and development of strategic emerging industries as a focal point for the transformation of the economic development mode. The selection of pilot cities to promote the integration of technology and finance is based on the *Notice on the Issuance of the Pilot Implementation Plan to Promote the Integration of Technology and Finance* issued by the Ministry of Science and Technology, the People’s Bank of China, the China Banking Regulatory Commission, the China Securities Regulatory Commission, the China Insurance Regulatory Commission, and the pilot program to promote the integration of technology and finance proposed by the regions. Eventually, 16 regions containing a total of 41 cities were identified as the first pilot cities. The distribution of science and technology finance pilot cities is shown in Figure 2. However, financing constraints are an influencing factor for domestic enterprises to absorb technology from foreign enterprises [17], and government intervention provides guarantees in the development of high-tech by providing financial support [18]. STFP has a positive impact on optimizing the efficiency of resource allocation, improving innovation performance, and promoting the transformation and upgrading of industrial structures, but the environmental effects of promoting the combination of technology and finance remain to be tested. Generally, cities with fast technological progress, which have strong economies, also show higher levels of carbon emissions [19,20]. Under the current carbon peak and carbon neutral targets, it is especially important to explore multiple ways to reduce carbon emissions; therefore, this paper focuses on whether the Science and Technology Finance Policy (STFP) can achieve the synergistic effect of reducing CO_2_ and play a radiating role in neighboring cities while fostering and developing strategic emerging industries, which are certainly of great practical importance. So, can STFP reduce carbon emission intensity in pilot cities? If so, how can this be achieved?

This paper uses the panel data of 274 cities in China from 2006 to 2017 to test the carbon emission reduction effect of STFP through the difference-in-differences method and further explores its transmission mechanisms and spatial effects. We focus on the impact of STFP on the carbon emission intensity of pilot cities and its realization path. We further investigate whether STFP and other related policies jointly reduce carbon emission intensity and explore whether the pilot policy creates a radiation effect, that is, whether it has a suppressive effect on carbon emission intensity in the surrounding areas. Therefore, this paper explores the main hypothesis: STFP has a suppressive effect on the carbon emission intensity of pilot cities. In addition, it is argued that the policy effect is mainly realized through three paths: total factor productivity, innovation factor agglomeration, and industrial structure. The main marginal contributions of this paper are as follows: (1) Along with the proposal of carbon neutrality, it is particularly important to seek ways of reducing carbon emissions without compromising economic development. This paper examines the carbon emission reduction effect of STFP, providing an important way of transforming and upgrading the economic development mode as well as achieving carbon neutrality. (2) This paper explores the dynamic effect of STFP on carbon emission intensity and judges whether the policy has a long-term impact on carbon emission intensity. Moreover, we further examine the transmission mechanisms of STFP on carbon emission reduction. (3) This paper examines the interactive effect between science and technology finance pilot cities and national e-commerce demonstration cities; this paper also explores whether the implementation of STFP can bring about significant spatial spillover effects. Thus, we not only explore the local carbon emission reduction effect of STFP but also further examine its impact on the carbon emission intensity in the surrounding areas.

## 2. Literature Review

### 2.1. The Effects of STFP

Accelerating the integration of technology and finance, cultivating, and developing strategic emerging industries, and then reducing carbon emissions will not only promote the transformation of economic development but also help achieve carbon peaking and carbon neutrality goals.

Academia has carried out research on the relationship between financial development and technology innovation for a long time. Schumpeter (1934) and Hicks (1969) believed that a large number of financial activities have a significant positive impact on technology innovation [21,22]. Rural financial development also contributes to agricultural technology innovation [23]. Thus, financing constraints have an inhibitory effect on enterprise innovation activities [24,25,26]. Different financing methods have different impacts on the technology innovation of enterprises; Hsu et al. (2014) studied 32 developed and emerging countries and found that the good development of the stock market helps those relying on external financial and high-tech-intensive industries to carry out innovative activities, while the development of the credit industry does not have a positive impact [27]. Furthermore, Kim et al. (2016) found that since bank loans require collateral, making managers more conservative and reluctant to invest in technology innovation activities, security issuance is more likely to promote the technological innovation of Korean listed companies than bank loans [28]. In addition, there is heterogeneity in the impact of financing constraints on firms’ technology innovation, with private firms found to be the most affected, followed by foreign, state-owned, and collective firms [29]. Political connections help mitigate the impact of financing pressure on private firms’ innovation performances [30]. Many scholars argue that there is an inverted U-shaped relationship between finance and innovation, which only promotes technology innovation to a certain extent [31,32]. Trinugroho et al. (2021) also believed that only when the development of credit and stock markets reach a certain threshold can they have a positive impact on the country’s innovation [33]. Interestingly, Liao (2020) found that substantive eco-innovation promotes corporate financing [34].

Later, scholars began to focus on the impact of the integration of technology and finance on green development as well as economic and social. In the long run, a poor financial environment not only negatively affects national innovation performance [35] but also exacerbates environmental degradation to some extent [36], while improved innovation mitigates the inhibitory effect of financial development on the environment. When exploring the relationship between financial development and green technology innovation, Lv et al. (2021) found that the banking-dominated financial structure significantly promoted green technology innovation, while the financial scale and financial efficiency inhibited green technology innovation [37]. The financing pressure of enterprises seriously restricts green technology innovation, and the negative impact on private enterprises is particularly significant, while green financial policies effectively alleviate the financing constraints of enterprises’ green technology innovation [38]. Due to the high cost of financial technology, the positive effect of green finance on smart city construction has a certain lag [39]. Hamberg (1966) studied the enterprises funded by the US Defense Department and found that the science and technology finance policy has a positive impact on the innovation investment of enterprises [40]. Sheng et al. (2021) found that the government’s financial investment in science and technology finance significantly promoted the innovation efficiency of the marine industry, while the effect of corporate capital investment was positive but not significant [41]. The digital economy, bank financing, and financial risks are all important factors that affect technology innovation, and the way to cultivate technology through the digital economy is particularly important [42]. In China, there is significant regional heterogeneity in sci-tech collaborative efficiency, and human capital and financing are important channels for promoting the integration of technology and finance [43]. Additionally, the development of science and technology finance has a positive impact on the coupling coordination between the technology transfer of universities and high-tech industries [44].

Thus, this paper proposes Hypothesis 1:

**H1.**  *STFP has a significant positive impact on carbon emission reduction in pilot cities.*

### 2.2. The Transmission Mechanisms

This paper believes that the impact of STFP on carbon emission intensity in pilot cities may come from the following aspects.

(1) Financial development serves to coordinate resource allocation and plays a positive role in promoting social innovation, and social financial opportunities have great potential for development [45]. Poor capital markets have a negative impact on innovation and economic growth, while government policies complement capital markets and relieve firms from external financing pressures [46]. Developmental financial institutions guide funds to invest in technology innovation, and financial support for technology innovation helps to compensate for market failures [47]. Therefore, the financial system not only provides funds for innovation activities and spreads risks but also has a good financial system that enhances the probability of innovation success and promotes economic growth [48,49]. This paper believes that the STFP optimizes resource allocation, enhances total factor productivity, and then reduces carbon emission intensity in pilot cities.

(2) R&D personnel and capital significantly promote innovation efficiency, which produces positive spatial spillover effects. The agglomeration of R&D personnel has a positive knowledge spillover effect and promotes technology progress through imitation, driving, and incentives, which in turn, has a significant positive effect on reducing the intensity of urban pollution emissions and improving environmental performance [50,51]. The *Several Opinions on Promoting the Integration of Science, Technology, and Finance to Accelerate the Implementation of Independent Innovation Strategy* proposes the cultivation of compound talents in technology and financial innovation and attracts high-end talents into the field of innovation and entrepreneurship. This paper believes that the STFP promotes the agglomeration of innovation elements, alleviates financing difficulties for enterprises, and, thus, achieves carbon emission reduction in pilot cities.

(3) The transformation of industrial structure can transform the impact of economic development on pollution [52], which is the main way to reduce energy intensity and carbon emissions [53,54,55]. The synergistic effect generated by technology innovation has a positive impact on enterprise productivity, which promotes the transformation of industrial structures [56,57]. The *Several Opinions on Promoting the Integration of Science, Technology, and Finance to Accelerate the Implementation of Independent Innovation Strategy* propose that promoting the combination of technology and finance is important for the strategic adjustment of economic structures, which is conducive to fostering strategic and emerging industries and helping the transformation and upgrading of industrial structures. This paper believes that the STFP promotes high-tech industries, optimizes the industrial structure, and reduces carbon emission intensity.

Thus, this paper proposes Hypothesis 2:

**H2.**  *STFP reduces carbon emission intensity in pilot cities through the total factor productivity enhancement effect, innovation elements agglomeration effect, and industrial structure optimization effect.*

## 3. Methodology and Data

### 3.1. Benchmark Model

According to the Notice on the Issuance of the Pilot Implementation Plan to Promote the Integration of Technology and Finance issued by the Ministry of Science and Technology, the People’s Bank of China, the China Banking Regulatory Commission, the China Securities Regulatory Commission, and the China Insurance Regulatory Commission, combined with the pilot programs for promoting the integration of technology and finance proposed by various regions, the Notice on the Identification of the First Batch of Pilot Areas for Promoting the Integration of Technology and Finance, issued on October 20, 2011, specifies that 16 regions containing 41 cities will take the lead in carrying out the pilot work. In June 2016, the second batch of 9 pilot cities was identified. To ensure the reliability of the results of the policy effect test, the STFP is considered a quasi-natural experiment to examine the carbon emission reduction effects generated by the first 41 pilot cities. Finally, 274 cities from 2006 to 2017 were selected as a panel data sample for the study. Among them, 41 pilot cities were the experimental group, and the remaining cities were the control group. The benchmark model is set as follows:

(1) $${CO}_{2}{}_{it}{=\alpha }_{0}{+\beta }_{t}\left(treat_{i}\times year_{t}\right){+\beta }_{x}X_{it}{+\delta }_{t}{+\mu }_{i}{+\varepsilon }_{it}$$

where ${CO}_{2}{}_{it}$ denotes the carbon emission intensity of *i* city in *t* year. The grouping dummy variable $treat_{i}\;$divides the sample data into the experimental group and control group. If the city is determined to be a science and technology finance pilot city, the value equals 1; otherwise, it is 0; The time dummy variable $year_{t}$ equals 0 for every year before 2012; otherwise, it is 0. $X_{it}\;$represents a series of control variables. $\alpha_{0}$ and $\beta_{t}$ denote the intercept term and estimated coefficient of the key independent variable, respectively. $\delta_{t}$ is the year fixed effect, $\mu_{i\;}$is the city fixed effect, and $\varepsilon_{it}$ is the random error term.

To test the dynamic policy effects generated by the STFP, this paper refers to the design of Jacobson et al. (1993) and Jia (2014) and adds the time trend into the benchmark model [58,59]. The specific model is set as follows:

(2) $${CO}_{2}{}_{it}{=\alpha }_{0}\;+\;\sum_{t\;=\;2012}^{t\;=\;2017}\beta_{t}\left({treat}_{i}{\times year}_{t}\right){\;+\;\beta }_{x}X_{it}{\;+\;\delta }_{t}{\;+\;\mu }_{i}{\;+\;\varepsilon }_{it}$$

### 3.2. Variable Selection and Data

(1) Dependent variable: carbon emission intensity (lncb) is represented by the natural logarithm of the ratio of total carbon dioxide emissions to the gross domestic product (GDP) [60]. The carbon emission data adopts the carbon dioxide emission data of China’s county-level cities calculated by Chen et al. (2020) [61] and aggregates the data of each county-level city into the corresponding prefecture-level cities, thus obtaining the carbon emission data of 274 prefecture-level cities.

(2) Key independent variable: $treat_{i}\times year_{t}$ represents the key independent variable ty, meaning the policy effects of STFP on carbon emission intensity.

(3) Control variables: In this paper, 6 control variables were selected: economic development, human capital, urbanization, financial correlation rate, education degree, and government intervention. Economic development (lngdp) is characterized by the natural logarithm of GDP [62]. Human capital (hum) is represented by the ratio of the number of employees in the technology and financial industries to the total number of employees at the end of the year [63]. Urbanization (urban) is expressed by the proportion of the population of the municipal district to the total population of the city [64]. The financial correlation ratio (finan) is defined as the ratio of the balance of deposits and loans of financial institutions to GDP [63]. Educational degree (edu) is measured by the ratio of the number of students in colleges to the total population [65]. Government intervention (inter) is represented by the proportion of government fiscal expenditure to the GDP [62].

(4) Mediating variables: this paper selects total factor productivity, innovation element agglomeration, and advanced industrial structure as intermediate variables. According to the design of Chung et al. (1997) [66], the total factor productivity (tfp) is calculated by MaxDEA8.0 software. The DDF-GML method is used to measure the total factor productivity index MI for each city using two types of indicators: input and output, where the input indicators include capital and labor, and the capital indicator uses the perpetual inventory method to calculate the actual capital stock of cities, and the labor indicator is characterized by the number of people employed at the end of the year in each city; the output indicator is the GDP of each region. In this paper, the total factor productivity in the base period of 2003 was set to 1, then the total factor productivity in 2004 was 1 multiplied by the MI in 2003, and so on, to calculate the total factor productivity of each city in each year. Based on the measurement method by Ciccone (2002) [67], this paper uses the employment density of employees in the scientific research and technology service industry to characterize the innovation elements agglomeration (sci). The advanced industrial structure (ais) is expressed by the ratio of the output value of the tertiary industry to that of the secondary industry [68].

This paper selects the panel data of 274 cities in China from 2006 to 2017. In addition to the CO_2_ data, the rest of the raw data were provided by the *China Urban Statistical Yearbook*. Descriptive statistics of the sample data are shown in Table 1.

## 4. Results and Discussion

### 4.1. The Effect of STFP on Carbon Emission Reduction

Table 2 shows the benchmark regression results of the impact of STFP on carbon emission intensity. In column (2), after adding the control variable, the coefficient of ty is still significantly negative at 1%, which means that the STFP has a significant inhibitory effect on the carbon emission intensity in pilot cities, and H1 is verified. STFP not only achieves the improvement of financial systems and technological capabilities but also reduces the carbon emission intensity of pilot cities, which helps to achieve the compatible development of the economy and the environment. Column (3) shows the time trend of the policy effects produced by STFP, and the policy coefficients show a trend that increases year by year. The inhibitory effect of STFP on the carbon emission intensity gradually changes from −0.024 in 2012 to −0.092 in 2017, and the significance degree increases from 10% to 1%, showing that STFP has a sustained dynamic effect; that is, it has a long-term suppressive effect on carbon emission intensity in pilot cities. With the implementation of STFP, the pilot areas have received financial incentives and support for technology innovation under the guidance of the policy. Furthermore, supportive policies have been improved, and the efficiency of resource allocation within the region has been optimized, creating a favorable environment for science and technology innovation, and vigorously cultivating strategic emerging industries, thus promoting the carbon emission reduction effect in pilot cities. Column (4) shows the result of the policy effect under the control of the parallel trend. The coefficients of ty are not significant before 2012, indicating that there was no significant difference between the experimental group and the control group before the implementation of STFP, which satisfies the parallel trend hypothesis. After the implementation of STFP, the policy effects gradually changed from −0.031 to −0.099, and the significance degree reached 1% in 2013. In conclusion, the policy effects of STFP on carbon emission reduction show a lasting dynamic effect that increases year by year. The STFP requires the vigorous guidance of the government to create a good financing environment for enterprises, promote the transformation of innovation achievements, and achieve a win-win situation of high-quality economic development and environmental dividends.

From the regression results with the control variables added, the coefficient of economic development on carbon emission intensity is significantly negative at 1%, indicating that high-quality economic development helps to reduce carbon emission intensity. The extensive economic development model in the past has not only met the requirements of high-quality development but has also resulted in tightening resource constraints and a decline in environmental quality, damaging the health of residents. High-quality economic development promotes the rational allocation of resources and improves total factor productivity but also gains environmental dividends. The coefficient of urbanization on carbon emission intensity is significantly negative at 10%, indicating that the urbanization process has an inhibitory effect on carbon emission intensity. The development of urbanization helps residents to accept new life concepts, pay more attention to environmental protection and low-carbon development, and promote the reduction in carbon emission intensity. The coefficients of the financial correlation rate and education degree on carbon emission intensity are significantly positive at 1%, meaning that the deposit and loan balances of financial institutions and education degrees show a positive relationship with carbon emission intensity. The coefficient of government intervention in carbon emission intensity is significantly positive at 5%. Due to the promotional needs of government officials, they often do not make great efforts to undertake environmental governance work and tend to sacrifice long-term environmental benefits to obtain short-term returns. Human capital does not have a significant impact on carbon intensity.

### 4.2. Event Study Method

**Figure 3:**
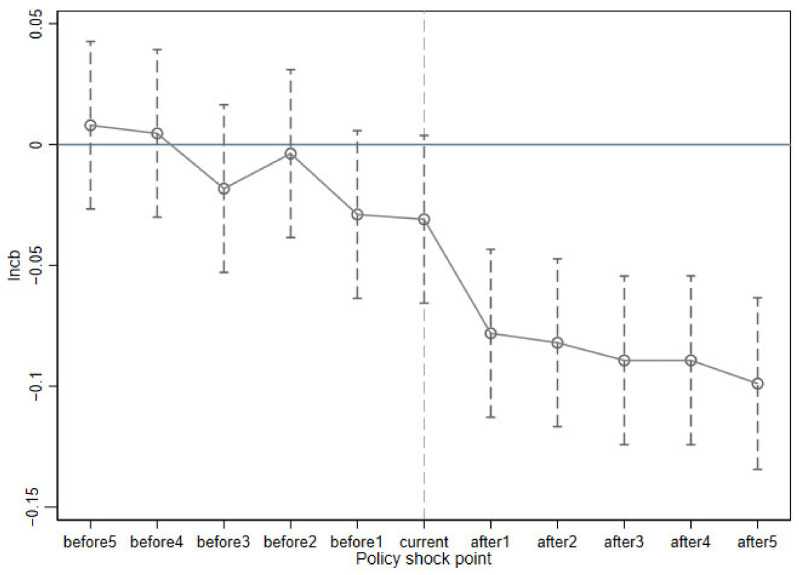
Event study method.

The premise of the difference-in-differences method is to satisfy the parallel trend assumption; that is, there is no significant difference between the treatment group and the control group before the policy occurs. If the treatment and control groups differ significantly before the implementation of the pilot policy, the policy effect assessed by the difference-in-differences method is biased. Therefore, this paper refers to the event study method proposed by Jacobson (2002) to test the parallel trend [69]. It can be seen from Figure 3 that the coefficients of ty before 2012 are near 0, which means there is no significant difference in carbon emissions between the treatment group and the control group before STFP, satisfying the parallel trend. After the implementation of STFP, a significant carbon emission reduction effect was produced, realizing the environmental improvement effect.

### 4.3. Heterogeneity Analysis

#### 4.3.1. Heterogeneous Effect of City Rank

It has been confirmed above that the STFP significantly suppresses carbon emission intensity in the pilot cities. Considering that the policy effects may be influenced by the city development level, this paper divides the sample into first and second-tier cities (RH) and other cities (RL) by referring to the *Ranking of Cities’ Business Attractiveness in China 2020*. When examining the impact of first and second-tier science and technology finance pilot cities on carbon emission intensity, set RH = 1 and RL = 0. When examining the impact of science and technology finance pilot cities in the third, fourth, and fifth tiers on carbon emission intensity, set RH = 0 and RL = 1.

The results in Table 3 show that the coefficient of STFP on carbon emissions in the first and second-tier cities is significantly negative at 1%, which means that STFP has a positive impact on carbon emission reduction in first and second-tier cities. First and second-tier cities have stronger financial support and high-tech talent, a more complete financial structure system, and a high level of technological innovation, so the implementation of STFP in such cities has a more significant policy effect. The combination of finance and technology helps cultivate strategic emerging industries, relieve financial pressure for enterprises, and promotes the improvement of environmental quality while realizing the transformation of economic development. In contrast, the economic development vitality of third, fourth, and fifth-tier cities is not strong enough, and the efficiency of urban resource allocation has yet to be optimized. A large amount of financial policy support and scientific research personnel are needed to promote the coordinated development of technology and finance. Thus, the implementation of STFP in such cities requires more financial preferential support and investment in technological innovation to create a favorable environment for financial development and innovation, and it will take a longer time to reverse the disadvantaged situation of distorted resource allocation and insufficient innovation momentum.

#### 4.3.2. Heterogeneous Effect of Information Level

This paper uses the ratio of the number of international Internet users to the total regional population at the end of the year to measure the urban information level. According to the average value of the information level, this paper divides the sample cities into the low information group (IL) and high information group (IH) and further explores the different impacts of STFP on carbon emission intensity in cities with different information levels. When exploring the impact of STFP on carbon emission intensity in cities with low information levels, set IL = 1 and IH = 0. When exploring the impact of STFP on carbon emission intensity in cities with high information levels, set IL = 0 and IH = 1.

The regression results in Table 4 show that the implementation of STFP in cities with higher information levels produces a more significant carbon emission reduction effect, while the STFP in cities with lower information levels does not produce a significant carbon emission reduction effect. Cities with high information levels have well-equipped infrastructure, high efficiency in technological innovation, and a favorable environment for innovation and development, which lays an advantageous foundation for the combination of technology and finance. However, cities with low information levels have weak financial development vitality, insufficient innovation power, and low sci-tech collaborative efficiency, requiring stronger policy guidance and financial support. Therefore, the STFP produces better carbon emission reduction effects in cities with higher information levels. By attracting preferential policies and creating a good innovation environment, the implementation of STFP promotes strategic emerging industries and fosters new economic growth points, thereby improving environmental quality and reducing carbon emission intensity.

## 5. Robustness Test

### 5.1. PSM-DID Test

To better identify the policy effects, this paper uses treat as the dependent variable and matches the science and technology finance pilot cities with the control group through radius matching and kernel matching methods. The PSM-DID examines the endogeneity problem caused by the self-selection bias of STFP in setting up pilot cities. The PSM-DID regression results in Table 5 show that the key independent variables are all significantly negative at 1%, which indicates that STFP does indeed reduce the carbon emission intensity of pilot cities and there is no obvious selection bias.

### 5.2. Control Other Policies

In the process of implementing STFP, there are other policies that interfere with the assessment of carbon emission reduction effects in this paper. The implementation of the low carbon city pilot policy, carbon emission trading (CET) policy and eco-civilized city alleviates environmental pollution and reduces carbon dioxide emissions to a certain extent, which affects the carbon emission reduction effect test of STFP. Thus, we further and respectively added the above policy interaction terms (the product of the grouping dummy variable and the time dummy variable of policy shock) as control variables in the benchmark model to exclude an estimation bias. The results in Table 6 show that after excluding the interference of the above three policies, the coefficients of ty were still significantly negative at 1%, indicating the STFP significantly reduces the carbon emission intensity in pilot cities, confirming the robustness of the previous benchmark regression results.

### 5.3. Sample Processing

In order to exclude the estimation bias caused by differences in regional economic levels, this paper excludes the samples of Beijing, Tianjin, Shanghai, and Chongqing. In addition, to overcome the problems of extreme data values and the policy effect estimation bias caused by the long sample interval, this paper also performs a 1% tail reduction for continuous variables in the sample data and shortens the data sample interval. The results in Table 7 show that the coefficients of the key independent variables are still significantly negative at 1%, indicating that the previous results are reliable after a series of robustness tests.

### 5.4. Placebo Test

**Figure 4:**
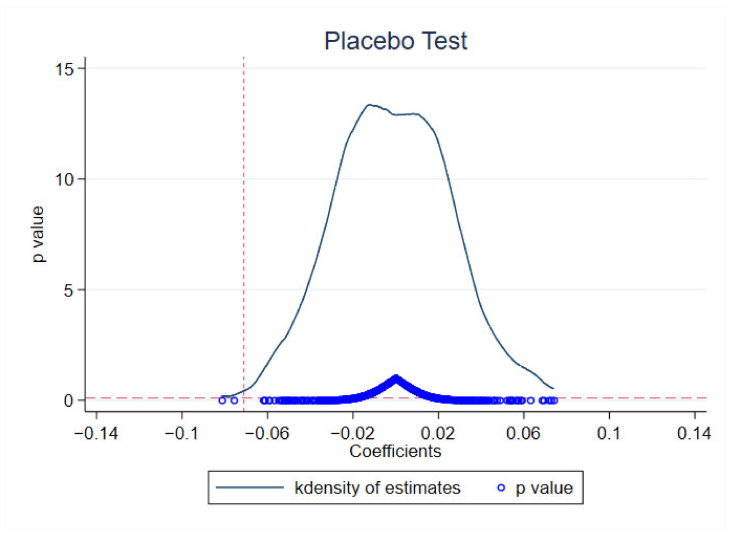
Placebo test.

To exclude the interference of other random factors, this paper also used a placebo test to verify the robustness of the baseline regression results. A total of 41 cities were randomly selected from 274 cities as the treatment group, and the remaining cities were the control group. In addition, to exclude other small probability events, this paper conducted 500 random samplings, and the mean of the estimated coefficients and p values are shown in Figure 4 below. The red dashed line represents the coefficient of the key independent variable in the baseline regression of this paper (−0.071). The placebo test results show that the estimated coefficients are all distributed around 0, and most of the p values are greater than 0.1, indicating that the placebo tests that randomly select pilot cities of STFP do not have an impact on carbon emission intensity, which in turn confirms the robustness of the baseline regression results.

## 6. Transmission Mechanisms

The above has confirmed that STFP has a positive impact on carbon emission reduction. So, what kind of path does the STFP take to reduce carbon emission intensity in the pilot cities? Referring to the research designs of Papyrakis and Gerlagh (2004) [70], this paper examines the impact of the total factor productivity improvement effect, innovation elements agglomeration effect, and industrial structure optimization effect on the carbon emission reduction in STFP:

(3) $$Med_{it}=\alpha_{0}+\varphi_{i}\left(treat_{i}\times year_{t}\right)+\beta_{x}X_{it}+\delta_{t}+\mu_{i}+\varepsilon_{it}$$

(4) $$CO_{2}{}_{it}=\alpha_{0}+\gamma_{i}\left(treat_{i}\times year_{t}\right)+\theta_{i}Med_{it}+\beta_{x}X_{it}+\delta_{t}+\mu_{i}+\varepsilon_{it}$$

where Med denotes the transmission mechanisms of STFP affecting carbon emission intensity. $\varphi_{i}\;$represents the effect of STFP on mechanism variables. $\theta_{i}\;$represents the effect of mechanism variables on carbon emission intensity. $\gamma_{i\;}$represents the effect of STFP on carbon emission intensity after adding the mechanism variables. Other variables are set to be consistent with Formula (1), and the regression results of the transmission mechanisms are shown in Table 8.

Columns (1) and (3) show that the coefficients of ty are significantly positive at 1%, indicating that the STFP has significantly improved the total factor productivity and innovation element agglomeration in pilot cities. Columns (2) and (4) show that the total factor productivity and innovation element agglomeration significantly suppress carbon emission intensity. It can be seen that the STFP curbs urban carbon emission intensity by the total factor productivity improvement effect and the innovation elements agglomeration effect. The science and technology finance pilot cities have more favorable financial development support as well as a technology innovation atmosphere to achieve the optimal allocation of resources and attract capital investment and R&D personnel, which is conducive to providing full play to various resources such as human, financial, and material advantages while promoting the coordinated development efficiency of technology and finance. Column (5) shows that the coefficient of ty is significantly positive at 10%, indicating that the STFP promotes the optimization of the industrial structure in pilot cities, and column (6) shows that the advanced industrial structure significantly reduces the carbon emission intensity. Promoting the combination of technology and finance eases the pressure of enterprise financing, guides the flow of capital to strategic emerging industries, and promotes scientific and technological research and development as well as the transformation of innovation results, which helps the transformation and upgrading of the industrial structure.

In conclusion, STFP achieves carbon emission reduction in pilot cities through the total factor productivity improvement effect, innovation elements agglomeration effect, and industrial structure optimization effect; thus, H2 is verified.

## 7. The Interactive Effect and Spatial Effect of STFP

### 7.1. Interactive Effect

In order to enhance the city’s economic influence and promote strategic emerging industries, the General Office of the State Council issued the *Guidance on the Establishment of National E-commerce Demonstration Cities* in 2011. In November 2011, the National Development and Reform Commission established 23 national e-commerce demonstration cities. Science and technology finance pilot cities and national e-commerce demonstration cities are important measures to realize the rational allocation of resources, optimize the industrial structure, and promote the modernization of the modern market system. Therefore, can STFP and national e-commerce demonstration cities achieve an interactive effect and jointly help cities to reduce their carbon emission intensity? Based on the above analysis, this paper constructs the following model:

(5) $${CO}_{2}{}_{it}{=\alpha }_{0}{+\beta }_{i}\left(treat_{i}\times year_{t}\times electronic_{i}\right){+\beta }_{x}X_{it}{+\delta }_{t}{+\mu }_{i}{+\varepsilon }_{it}$$

where $electronic_{i}$ denotes the grouping dummy variable of national e-commerce demonstration cities; if the city belongs to the 23 national e-commerce demonstration cities, it is one; otherwise, it is zero, and the rest of the variable selection and model settings are consistent with Formula (1).

Table 9 shows the interactive effect test between the STFP and the national e-commerce pilot policy. It can be seen from columns (1) and (2) that the coefficients of the triple interaction items are always significantly negative at 1%. Interestingly, the triple interaction coefficient (−0.157) is greater than the coefficient of ty (−0.071) in Table 2, indicating that the establishment of national e-commerce pilot cities has a positive impact on the carbon emission reduction effect of STFP, and the two have an interactive effect, jointly reducing the urban carbon emission intensity. The national e-commerce demonstration city is similar to STFP: they inject new vitality into economic development by promoting the development of strategic emerging industries, improving the efficiency of resource allocation, and enhancing the city’s core competitiveness. Both of these help reduce the consumption of material resources and energy, alleviate environmental pollution problems, and reduce carbon emissions, which is an important path to achieving carbon neutrality.

### 7.2. Spatial Effect

The above proves that STFP has a positive carbon emission reduction effect. Since the pilot policy can significantly reduce the local carbon intensity, the question is, will it have an impact on the surrounding areas? Referring to the experimental design of Jiang et al. (2021) [71], this paper constructs the difference-in-differences model again to explore the spatial effect of STFP:

(6) $${\;CO}_{2}{}_{it}{=\alpha }_{0}+\gamma_{i}\left(near_{i}{\times \mathrm{year}}_{t}\right){+\beta }_{x}X_{it}{+\delta }_{t}{+\mu }_{i}{+\varepsilon }_{it}$$

where $near_{i}$ is the grouping variable bordering the pilot cities of STFP. The non-pilot cities bordering the pilot cities are set as the experimental group, and the grouping dummy variable is set to one; otherwise, it is set to zero. Other variable settings remain the same as the benchmark model.

The results in Table 10 show that no matter whether the control variables are added or not, the interaction coefficients are still significantly negative at 1%, which is consistent with the direction of the benchmark regression, indicating that the implementation of STFP reduces the carbon emission intensity of the neighboring areas. It can be seen that the carbon emission reduction effect of STFP has a significant radiation effect; that is, it not only reduces the local carbon emission intensity but also significantly inhibits the carbon emission intensity of the neighboring areas. By promoting the combination of technology and finance, the pilot cities not only have a sound financial structure system but also have a good innovation atmosphere, playing an exemplary leading role for neighboring areas. While promoting the coordinated and rapid development of technology and finance in pilot cities has a significant spatial spillover effect, driving the combined development of technology and finance in the surrounding cities and not only cultivating new economic growth points but also achieving the carbon reduction in the surrounding areas.

## 8. Conclusions

Based on the panel data of 274 cities in China from 2006 to 2017, this paper explores the impact of STFP on carbon emission intensity through the difference-in-differences method and further tests the transmission mechanisms. Considering the possible impact of the science and technology finance pilot policy on the surrounding areas, this paper also examines the spatial effects of STFP. Interestingly, we found that STFP had a significantly positive impact on the carbon emission reduction in the surrounding cities and created an interactive effect with national e-commerce demonstration cities. The main conclusions are shown in Table 11, specifically:

(1) STFP significantly reduces local carbon emission intensity and has a continuous dynamic effect. With the implementation of the pilot policy, the carbon emission reduction effect shows an increasing trend year by year. The government should continue to implement the measures related to the pilot project to promote the integration of science, technology, and finance to realize the synergistic effect of carbon emission reduction and jointly contribute to the realization of carbon neutrality.

(2) First and second-tier cities have a higher financial development level and are conducive to promoting resource allocation efficiency. In addition, cities with a high information level provide a solid foundation for the development and cultivation of new technologies. Therefore, the STFP is implemented effectively in these two types of cities, which helps to vigorously cultivate strategic emerging industries and reduce carbon emission intensity. A high degree of integration in terms of technology and finance can help reduce carbon emission intensity.

(3) The total factor productivity improvement, innovation factor concentration, and optimization of industrial structures are the main paths of STFP to achieve carbon emission reduction. The government should alleviate the financial difficulties of new technological development through financial policy support, the improvement of independent innovation capacity and productivity, and the vigorous cultivation of strategic and emerging industries, thus reducing carbon emission intensity in the pilot areas.

(4) STFP has an interactive effect with national e-commerce demonstration cities, and they jointly reduce carbon emission intensity in pilot cities. Considering the policy spatial effect, STFP reduces carbon emission intensity in local and neighboring areas producing a significant radiation effect. In addition to achieving the objectives of the policies themselves, the linkage effect between the policies should be actively explored to further realize the synergistic effect of carbon emission reduction and to serve as a model for other cities.

The policy implications of this paper are as follows: (1) due to the significant differences in technology and financial structure in different regions, the government should pay attention to the differences between cities when implementing policies to promote the integration of technology and finance and guide funds to flow into new technology fields with great development potential. Policy support for third, fourth, and fifth-tier cities and cities with low information levels should be increased to narrow the development gap between regions. (2) The total factor productivity improvement effect, the innovation elements agglomeration effect, and the industrial structure optimization effect are all important ways to reduce carbon emission intensity, but the industrial structure optimization effect generated by STFP is relatively small. The government should focus on financial support policies to alleviate the financial difficulties of high-tech enterprises, cultivate strategic emerging technologies, and accelerate the transformation and upgrading of industrial structures. (3) Attention should be paid to the interactive effect of policy dividends, and the reduction in carbon emissions should be promoted, thereby realizing environmental dividends. Other regions may learn from or imitate the policy experience of STFP, and each region should formulate relevant policies reasonably according to its own actual situation. Through the development of science and technology finance in pilot cities, the surrounding areas will be driven, and the radiation effect of policy dividends will be exerted.

## Figures and Tables

**Table 1:** Descriptive statistics.

Variable	Note	Definition	Mean	Min	Max
lncb	Carbon emission intensity	Natural logarithm of the ratio of total carbon emissions to GDP	7.5350	5.0488	9.3494
lngdp	Economic development	Natural logarithm of GDP	16.1759	13.1602	19.6116
hum	Human capital	Ratio of the number of employees in the technology and financial industries to the total number of employees	0.0267	0.0036	0.4243
urban	Urbanization	Ratio of the population of the municipal district to that of the city	0.3458	0.0436	1.0000
finan	Financial correlation ratio	Ratio of the balance of deposits and loans of financial institutions to GDP	2.0751	0.5600	12.5079
edu	Educational degree	Ratio of the number of students in colleges to the total population	0.0154	0.0000	0.1311
inter	Government intervention	Ratio of government expenditure to GDP	0.2068	0.0426	6.0406
inm	Information level	Ratio of international Internet users to total regional population	0.1474	0.0001	3.6635
tfp	Total factor productivity	Calculated through the DDF-GML model of the DEA method	1.7699	0.1834	12.6500
sci	Innovative elements agglomeration	Ratio of the number of R&D personnel to urban area	0.4757	0.0113	3.9631
ais	Advanced industrial structure	Ratio of the output value of tertiary industry to that of secondary industry	1.4458	0.1823	21.2601

**Table 2:** The impact of STFP on carbon emission.

	(1)	(2)	(3)	(4)
	lncb	lncb	lncb	lncb
ty	−0.135 ***	−0.071 ***		
	(0.011)	(0.007)		
ty07				0.008
				(0.018)
ty08				0.005
				(0.018)
ty09				−0.018
				(0.018)
ty10				−0.004
				(0.018)
ty11				−0.029
				(0.018)
ty12			−0.024 *	−0.031 *
			(0.014)	(0.018)
ty13			−0.072 ***	−0.078 ***
			(0.014)	(0.018)
ty14			−0.076 ***	−0.082 ***
			(0.014)	(0.018)
ty15			−0.083 ***	−0.089 ***
			(0.014)	(0.018)
ty16			−0.083 ***	−0.089 ***
			(0.014)	(0.018)
ty17			−0.092 ***	−0.099 ***
			(0.014)	(0.018)
lngdp		−0.723 ***	−0.719 ***	−0.717 ***
		(0.013)	(0.013)	(0.013)
hum		0.034	0.037	0.039
		(0.138)	(0.137)	(0.137)
urban		−0.064 *	−0.054	−0.051
		(0.034)	(0.034)	(0.034)
finan		0.042 ***	0.042 ***	0.042 ***
		(0.004)	(0.004)	(0.004)
edu		1.097 ***	1.104 ***	1.107 ***
		(0.320)	(0.319)	(0.320)
inter		0.021 **	0.018 **	0.018 **
		(0.008)	(0.008)	(0.008)
_cons	7.545 ***	19.149 ***	19.076 ***	19.048 ***
	(0.002)	(0.213)	(0.213)	(0.214)
*Time effect*	Yes	Yes	Yes	Yes
*City effect*	Yes	Yes	Yes	Yes
N	3288	3288	3288	3288
R−sq	0.962	0.985	0.985	0.985

Note: Standard errors are in parentheses, and ***, **, * denote significance at the levels of 1%, 5%, and 10%, respectively.

**Table 3:** Heterogeneity of city rank.

	(1)	(2)
	lncb	lncb
ty×RL	−0.001	
	(0.011)	
ty×RH		−0.110 ***
		(0.009)
lngdp	−0.738 ***	−0.733 ***
	(0.013)	(0.013)
hum	0.049	0.033
	(0.140)	(0.137)
urban	−0.107 ***	−0.056 *
	(0.034)	(0.033)
finan	0.041 ***	0.042 ***
	(0.004)	(0.004)
edu	1.082 ***	1.352 ***
	(0.325)	(0.318)
inter	0.027 ***	0.021 **
	(0.008)	(0.008)
_cons	19.404 ***	19.306 ***
	(0.216)	(0.209)
*Time effect*	Yes	Yes
*City effect*	Yes	Yes
N	3288	3288
R−sq	0.984	0.985

Note: Standard errors are in parentheses, and ***, **, * denote significance at the levels of 1%, 5%, and 10%, respectively.

**Table 4:** Heterogeneity of information level.

	(1)	(2)
	lncb	lncb
ty×IL	0.007	
	(0.013)	
ty×IH		−0.082 ***
		(0.008)
lngdp	−0.739 ***	−0.727 ***
	(0.013)	(0.013)
hum	0.050	0.039
	(0.140)	(0.137)
urban	−0.107 ***	−0.059 *
	(0.034)	(0.033)
finan	0.041 ***	0.042 ***
	(0.004)	(0.004)
edu	1.091 ***	1.169 ***
	(0.325)	(0.319)
inter	0.027 ***	0.019 **
	(0.008)	(0.008)
_cons	19.415 ***	19.209 ***
	(0.215)	(0.211)
*Time effect*	Yes	Yes
*City effect*	Yes	Yes
N	3288	3288
R−sq	0.984	0.985

Note: Standard errors are in parentheses, and ***, **, * denote significance at the levels of 1%, 5%, and 10%, respectively.

**Table 5:** PSM-DID test.

	Radius Matching	Kernel Matching
	(1)	(2)
	lncb	lncb
ty	−0.038 ***	−0.039 ***
	(0.008)	(0.008)
lngdp	−0.743 ***	−0.742 ***
	(0.013)	(0.013)
hum	0.050	0.051
	(0.132)	(0.132)
urban	−0.054	−0.053
	(0.033)	(0.033)
finan	0.040 ***	0.040 ***
	(0.004)	(0.004)
edu	1.170 ***	1.164 ***
	(0.317)	(0.316)
inter	0.020 **	0.020 **
	(0.010)	(0.010)
_cons	20.614 ***	20.592 ***
	(0.235)	(0.232)
*Time effect*	Yes	Yes
*City effect*	Yes	Yes
N	3160	3170
R−sq	0.984	0.984

Note: Standard errors are in parentheses, and ***, ** denote significance at the levels of 1% and 5%, respectively.

**Table 6:** Control other policies.

	Control Low Carbon City	Control Eco-Civilized City	Control CET
	(1)	(2)	(3)
	lncb	lncb	lncb
ty	−0.070 ***	−0.069 ***	−0.068 ***
	(0.008)	(0.007)	(0.007)
lngdp	−0.724 ***	−0.723 ***	−0.716 ***
	(0.013)	(0.013)	(0.013)
hum	0.032	0.041	0.060
	(0.138)	(0.138)	(0.136)
urban	−0.063 *	−0.060 *	−0.028
	(0.034)	(0.034)	(0.033)
finan	0.042 ***	0.042 ***	0.040 ***
	(0.004)	(0.004)	(0.004)
edu	1.128 ***	1.111 ***	1.142 ***
	(0.322)	(0.320)	(0.315)
inter	0.021 **	0.020 **	0.019 **
	(0.008)	(0.008)	(0.008)
_cons	19.157 ***	19.152 ***	19.025 ***
	(0.213)	(0.213)	(0.210)
*Time effect*	Yes	Yes	Yes
*City effect*	Yes	Yes	Yes
N	3288	3288	3288
R−sq	0.985	0.985	0.985

Note: Standard errors are in parentheses, and ***, **, * denote significance at the levels of 1%, 5%, and 10%, respectively.

**Table 7:** Sample processing.

	Remove Municipalities	Tail Shrinking Treatment	Shorten Sample Interval
	(1)	(2)	(3)
	lncb	lncb	lncb
ty	−0.053 ***	−0.059 ***	−0.057 ***
	(0.008)	(0.008)	(0.008)
lngdp	−0.723 ***	−0.685 ***	−0.811 ***
	(0.013)	(0.013)	(0.014)
hum	0.073	0.490 *	0.119
	(0.136)	(0.261)	(0.129)
urban	−0.061 *	−0.072 **	−0.063 *
	(0.033)	(0.034)	(0.034)
finan	0.045 ***	0.048 ***	0.030 ***
	(0.004)	(0.005)	(0.004)
edu	0.865 **	1.184 ***	1.356 ***
	(0.314)	(0.349)	(0.405)
inter	0.020 **	0.045 ***	0.004
	(0.008)	(0.015)	(0.007)
_cons	19.133 ***	18.507 ***	20.632 ***
	(0.209)	(0.220)	(0.239)
*Time effect*	Yes	Yes	Yes
*City effect*	Yes	Yes	Yes
N	3240	3288	2466
R−sq	0.985	0.983	0.988

Note: Standard errors are in parentheses, and ***, **, * denote significance at the levels of 1%, 5%, and 10%, respectively.

**Table 8:** Transmission mechanisms.

	(1)	(2)	(3)	(4)	(5)	(6)
	tfp	lncb	sci	lncb	ais	lncb
ty	0.869 ***	−0.047 ***	0.168 ***	−0.056 ***	0.092 *	−0.070 ***
	(0.044)	(0.008)	(0.010)	(0.008)	(0.051)	(0.007)
tfp		−0.027 ***				
		(0.003)				
sci				−0.089 ***		
				(0.013)		
ais						−0.008 ***
						(0.003)
lngdp	1.186 ***	−0.690 ***	0.015	−0.722 ***	−0.625 ***	−0.728 ***
	(0.077)	(0.013)	(0.018)	(0.013)	(0.089)	(0.013)
hum	9.312 ***	0.290 **	7.720 ***	0.719 ***	5.140 ***	0.077
	(0.825)	(0.139)	(0.192)	(0.170)	(0.951)	(0.138)
urban	1.404 ***	−0.025	0.258 ***	−0.041	−0.235	−0.066 **
	(0.201)	(0.033)	(0.047)	(0.033)	(0.232)	(0.033)
finan	0.076 ***	0.044 ***	−0.004	0.041 ***	0.014	0.042 ***
	(0.025)	(0.004)	(0.006)	(0.004)	(0.029)	(0.004)
hr	1.733	1.145 ***	5.785 ***	1.611 ***	9.934 ***	1.181 ***
	(1.915)	(0.315)	(0.445)	(0.326)	(2.208)	(0.320)
inter	−0.354 ***	0.011	−0.029 ***	0.019 **	0.057	0.022 ***
	(0.049)	(0.008)	(0.011)	(0.008)	(0.056)	(0.008)
_cons	−18.318 ***	18.646 ***	−0.152	19.136 ***	11.293 ***	19.245 ***
	(1.275)	(0.217)	(0.296)	(0.211)	(1.470)	(0.215)
*Time effect*	Yes	Yes	Yes	Yes	Yes	Yes
*City effect*	Yes	Yes	Yes	Yes	Yes	Yes
N	3288	3288	3288	3288	3288	3288
R−sq	0.806	0.985	0.970	0.985	0.804	0.985

Note: Standard errors are in parentheses, and ***, **, * denote significance at the levels of 1%, 5%, and 10%, respectively.

**Table 9:** The interactive effect of STFP.

	(1)	(2)
	lncb	lncb
$treat_{i}\times year_{t}\times electronic_{i}$	−0.174 ***	−0.157 ***
	(0.019)	(0.012)
lngdp		−0.731 ***
		(0.013)
hum		0.057
		(0.136)
urban		−0.042
		(0.033)
finan		0.043 ***
		(0.004)
hr		1.249 ***
		(0.316)
inter		0.023 ***
		(0.008)
_cons	7.539 ***	19.265 ***
	(0.002)	(0.209)
*Time effect*	Yes	Yes
*City effect*	Yes	Yes
N	3288	3288
R−sq	0.961	0.985

Note: Standard errors are in parentheses, and *** denote significance at the levels of 1%.

**Table 10:** The spatial effects of STFP.

	(1)	(2)
	lncb	lncb
$near_{i}{\times \mathrm{year}}_{t}$	−0.074 ***	−0.051 ***
	(0.010)	(0.006)
lngdp		−0.721 ***
		(0.014)
hum		0.080
		(0.142)
urban		−0.052
		(0.038)
finan		0.050 ***
		(0.005)
hr		0.907 **
		(0.359)
inter		0.012
		(0.009)
_cons	7.617 ***	19.041 ***
	(0.003)	(0.226)
*Time effect*	Yes	Yes
*City effect*	Yes	Yes
N	2796	2796
R−sq	0.956	0.983

Note: Standard errors are in parentheses, and ***, ** denote significance at the levels of 1%, 5%, respectively.

**Table 11:** Summary of results: five impacts of STFP on carbon emission intensity.

	Empirical Main Findings
Effect I: Direct effect	STFP has a significant positive impact on carbon emission reduction in pilot cities.
Effect II: Heterogeneous effects	STFP has a positive impact on carbon emission reduction in first and second tier cities.
	STFP produces a better carbon emission reduction effect in cities with higher information levels.
Effect III: Intermediary effects	STFP contributes to the reduction in carbon emission intensity by improving total factor productivity.
	STFP promotes the carbon emission reduction effect through the agglomeration of innovation elements.
	STFP reduces carbon emission intensity by optimizing the industrial structure.
Effect IV: Interactive effect	STFP and national e-commerce demonstration cities achieve an interactive effect and jointly help cities to reduce carbon emission intensity.
Effect V: Spatial effect	STFP has a significant radiation effect; that is, it not only reduces local carbon emission intensity but also inhibits the carbon emission intensity of neighboring areas.

## Data Availability

The datasets used and analyzed in the current study are available from the corresponding author upon reasonable request.
